# A survey to investigate the association of pain, foot disability and quality of life with corns

**DOI:** 10.1186/s13047-015-0131-4

**Published:** 2015-12-08

**Authors:** Lisa Farndon, Michael Concannon, John Stephenson

**Affiliations:** Jordanthorpe Health Centre, 1 Dyche Close, Sheffield, S8 8DJ UK; Department of Health Sciences, University of Huddersfield, Queensgate, Huddersfield, HD1 3DH UK

**Keywords:** Corns, pain, quality of life, foot disability

## Abstract

**Background:**

Corns are a common foot problem affecting a large proportion of the population. This study describes the characteristics of corns experienced by 201 participants taking part in a randomised controlled trial to investigate associations between demographic and corn parameters on pain, foot related disability and quality of life (QoL).

**Methods:**

Pain from the main (index) corn was measured using a visual analogue scale (VAS); foot related disability was assessed with the Foot Disability Questionnaire (now known as the Manchester Foot Pain and Disability Index) and quality of life was recorded with the EQ-5D questionnaire. The effect of demographic and corn parameters on the pain and quality of life outcomes was assessed with analysis of variance (ANOVA) methods. The effect of the same factors on a linear combination of the foot-related disability outcome measures was assessed using multivariate ANOVA methods. Pain was also tested for its mediating properties on the causal pathway between the independent variables and quality of life.

**Results:**

The mean pain score was 5.29 points on a 10 cm VAS, with females reporting substantively higher pain levels than males. Age affected foot-related disability, with lower levels on all domains of the MFPDI reported in older participants; each year of advancing age was associated with falls of: 0.009 points on the Concern about Appearance (CA) domain; 0.047 points on the Functional Limitation (FL) domain and 0.048 points on the Pain Intensity (PI) domain. Sex and corn type also affected disability, with higher scores reported by females and participants with plantar corns.

**Conclusions:**

The effect of pain was shown to mediate the relationship between sex and foot-related disability. The presence of plantar corns has a more detrimental effect on QoL than dorsal/inter-digital corns.

**Trial registration:**

ISRCTN 13166839

## Background

Foot problems and foot pain are common [1, 2], whilst corns can be found in 14–48 % of people [3]. Of 111 participants attending a podiatry clinic, nearly half (46 %) were found to have calluses and corns [4] and a review of 392 participants attending an Australian podiatry clinic also reported that the most commonly presenting problems were nails, corns and calluses [5]. Large epidemiological surveys also report a high prevalence of foot problems and pathologies: of 76,475 people attending a dermatology or general practice department, just over half (57 %) had a foot disease [6]. The most common were general skin conditions (eczema, psoriasis, fungal infections) and metatarsal corns. Foot problems are associated with pain and can affect quality of life. A large survey of 3206 people found that the 17 % who reported foot pain scored lower across all domains on a standard quality of life measure (SF-36) [7]. This foot pain was associated with increased age and was more common in women and those who were obese. A systematic review also found that women have higher incidences of foot/ankle pain which is age-related, and two-thirds of cases reported moderate disability as a result of this which affected daily life [8].

As outlined above, the association of foot pain and disability is a commonly recurring factor in many studies. ‘*Foot trouble*’ was the single most cited factor affecting activities of daily living in a small study of older people [9], and similar results have been found elsewhere in which a statistically significant association between foot problems and pain, and activities of daily living was identified [10]. Older women (65 years and older) have been found to suffer severe foot pain which is associated with difficulty in walking and affects daily living activities [11]. Foot pain and function are therefore strongly correlated [4].

Corn production is thought to be stimulated by trauma to the tissues in the form of mechanical stresses, which cause the release of inflammatory mediators and growth factors [12]. These chemical mediators are thought to increase cell production, transit time through the epidermis, and cohesion between the cells [12], resulting in either a plaque of callus or a corn. A corn is an area of callus moulded into a nucleus [13]. Pain caused by corns can be alleviated if a scalpel is used to remove the tissue [14]. Foot pain and corns are also associated with poorly fitting footwear. A survey of 176 older people found that foot pain, disability and corns were more common in those wearing narrower shoes [15].

Whilst foot problems, including corns, have been shown to be a common condition and can be associated with pain, much of the available survey data does not solely concentrate on this foot condition, but involves a range of pathologies. However, in a survey to determine the current participant population managed by UK podiatrists Farndon 2015 [16] reported that the most frequently presenting problems were corns and callus (26 %) resulting in this being the most common type of treatment performed by the profession (19 %) in a typical working day.

Corns are a common foot problem affecting a large proportion of the population. This study aims to quantify the effect of corn size and type, controlling for demographic factors, on pain, foot related disability and quality of life.

## Methods

As part of a randomised controlled trial to investigate the long term effectiveness of 40 % salicylic acid plasters for the treatment of corns [17], participants were surveyed to determine the pain and disability that they experienced from corns. The type, site and size of each corn were recorded by a podiatrist, along with the pain experienced from the main (index) corn. The foot-related disability and quality of life (QoL) were then documented. All participants included in the trial had one or more corns. Participants were recruited from those currently attending for podiatry treatment from within one NHS podiatry service, one university podiatry clinic and the local general population between September 2009 and October 2011. Participants were also recruited who were not currently receiving podiatry treatment via posters and leaflets in GP surgeries and health centres and advertisements placed in a local free paper. Participants were excluded if they: had diabetes or rheumatoid arthritis; had poor peripheral circulation or peripheral neuropathy; had a history of foot ulceration; were taking oral steroid medication; had a marked dermatological condition which affected skin texture in the feet (e.g. eczema, psoriasis); were allergic to zinc oxide plaster, salicylic acid, peanuts or soya; were unable to reach their own feet; had calluses rather than corns, or corns that were infected or neurovascular; or were pregnant or breastfeeding.

At the initial appointment the site, diameter (mm) and type (categorized into plantar and dorsal/inter-digital) of up to three corns per participant was recorded by a research podiatrist. If more than one corn was present, the participant was asked to nominate an index (main) corn which was used for analysis (second and subsequent corns recorded by each participant were not included in the analysis). The age and sex of each participant was also noted.

Participants were asked to indicate the pain they were experiencing from the index corn on a 10 cm Visual Analogue Scale (VAS), where 0 = no pain; 10 = worst possible pain. Each participant was then asked to complete the Foot Disability Questionnaire [18], now known as the Manchester Foot Pain and Disability Index (MFPDI) [19], and the EQ-5D quality of life questionnaire [20] to determine if the presence of corns was associated with pain, foot disability and affected quality of life (QoL). Lower VAS pain scores represent reduced pain; high MFPDI scores represent higher levels of foot disability; higher EQ-5D scores represent better QoL.

The current analysis is concerned with the baseline measurements reported in the clinical trial: further assessment of the longitudinal aspect of the study (post-randomisation) will be reported in subsequent publications.

### Statistical analysis

The sample was summarized descriptively. Exploratory data analyses were undertaken to check for collinearity among independent variables (age, sex, corn size and corn type), to verify the suitability of multivariate procedures, and to determine whether transformations of outcome measures (the MFPID subscales, the EQ-5D score and the VAS pain score) were required to achieve normality of residuals or to stabilize variance. Outcomes were assessed for suitability for multivariate or univariate treatment. A multivariate analysis was conducted on the data, considering initially all main effects and first-order interactions, to analyse the relationship between demographic factors (age, sex) and corn parameters (size, type); and foot-related disability outcomes, which comprised the functional limitation (FL), pain intensity (PI) and concern about appearance (CA) sub-scales of the MFPDI instrument. Items were assigned to the appropriate subscale using the factor structure devised by Garrow [18]. Follow-up univariate analyses were conducted where appropriate to investigate the source of statistically significant or substantive differences. Further univariate analyses were conducted on QoL (using the EQ-5D instrument), and pain, using the self-reported visual analogue scale (VAS); to assess the relationships between these outcomes and the same set of predictor variables.

Statistical significance was set at the 5 % level. Following standard procedures, non-significant interactions were removed from all models, which were then re-cast including main effects and any remaining interactions. Multivariate and univariate relationships were assessed using tests of significance and confidence intervals. Effect sizes of significant factors were assessed using the partial-η^2^ statistic. Regression assumptions were tested by examination of residual plots.

The pain outcome was also tested as a mediating variable on the causal pathway between the independent variables and the EQ-5D QoL outcome measure, using the criterion of Baron and Kenny [21] to assess mediation.

All statistical analysis was undertaken using SPSS (Version 20.0) statistical software.

## Results

### Descriptive and exploratory analysis

**Fig. 1:**
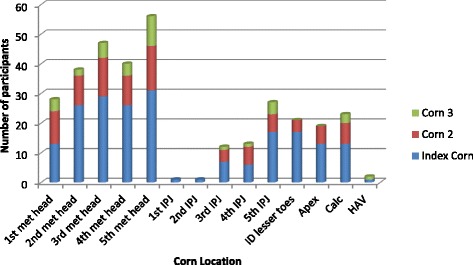
Corn Location. This figure illustrates the location of the index corn, second and third corns (if present), per participant. The location of corns were ordered via their anatomical site, e.g. metatarsal head (met head). Inter-phalangeal joint (*IPJ*), inter-digitally (*ID*), the apices of the toes (*apex*) and over a bunion (*HAV*)

Data was obtained from 201 participants. One hundred and fourteen participants had only 1 corn, 85 participants had 2 corns and 35 participants had 3 or more corns. The most commonly occurring sites for corns were the metatarsal heads, followed by the 5^th^ inter-phalangeal joint (dorsally) (Fig. 1). The demographics and outcome measures are summarised in Table 1.

**Table 1:** Summary of demographic and outcome measures

Categorical variable	Frequency (%)
Sex	
Males	84 (41.8 %)
Females	117 (58.2 %)
Corn type	
Plantar	151 (75.1 %)
Dorsal/inter-digital	50 (24.9 %)
Numerical variable	Mean (SD); range
Age (years)	59.3 (16.5); 19 to 90
Baseline corn diameter (mm)	3.8 (1.8); 1.0 to 10.0
MFPDI –FL subscale	18.0 (5.36); 10 to 30
MFPDI –PI subscale	8.96 (2.83); 5 to 15
MFPDI –CA subscale	3.41 (1.41); 2 to 6
EQ-5D QoL	73.9 (19.9); 0 to 100
VAS Pain	5.29 (2.97); 0 to 10

Exploratory analysis of outcome data revealed no evidence for collinearity between independent variables, and that all subscale readings of the MFPDI instrument were recorded across the full range of the respective scales. The CA subscale scores of the MFPDI instrument were found to exhibit positive skew and heterogeneity of variance of residuals. A log transformation was applied to this variable resulting in improved normality and homogeneity of variance. Statistically significant correlations were found to occur between each pair of MFPDI subscale scores, suggesting a requirement for a multivariate approach to the analysis. No corresponding requirement for multivariate treatment of the pain or QoL measures was revealed; hence these outcomes were analysed on a univariate basis. No requirement for data transformations with respect to the QoL or pain outcomes was found. Examination of residual plots did not reveal any violations of regression assumptions in any model.

### Analysis of foot disability scores

**Fig. 2:**
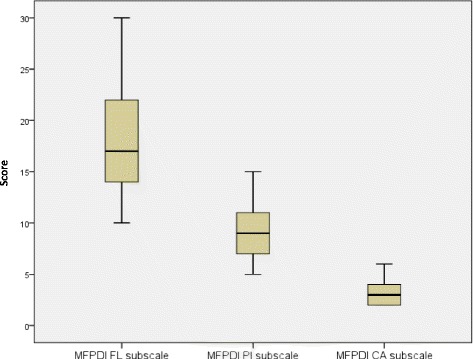
MFPDI subscale score distributions. This figure depicts the mean scores of the three subscales of the Manchester Foot Pain and Disability Index; Functional Limitation (*FL*), Pain Intensity (*PI*) and Cosmetic Appearance (*CA*)

The mean scores of the FL, PI and CA subscales of the MFPDI instrument were, respectively, 18.0 (SD 5.36); 8.96 (SD 2.83) and 3.41 (SD 1.40); summarised graphically in Fig. 2.

A main effects analysis on a linear combination of the MFPDI subscale scores found age to be significantly associated with a linear combination of the outcome measures (Λ = 0.837; *F*_3,188_ = 12.2; *p* < 0.001). The partial-η^2^ statistic of 0.163 indicated a medium effect.

Follow-up univariate analyses indicated age to be significantly associated with all three outcome measures. The association was most pronounced on the PI subscale (*F*_1,190_ = 16.2; *p* < 0.001); and the transformed CA subscale (*F*_1,190_ = 25.9; *p* < 0.001). The association with the FL subscale was slightly less pronounced, exhibiting borderline statistical significance (*F*_1,190_ = 3.93; *p* = 0.049). Each year of increasing age was associated with a mean reduction of 0.048 points on the PI subscale, a mean reduction of 0.047 points on the FL subscale, and a mean reduction of 0.009 points on the CA subscale after an exponential back-transformation.

Sex and corn type were both found to exhibit some substantive association with a linear combination of the outcome measures, although these associations were not statistically significant at the 5 % significance level (Λ = 0.966; *F*_3,188_ = 2.20; *p* = 0.090 for sex: Λ = 0.969; *F*_3,188_ = 2.01; *p* = 0.114 for corn type). No statistically significant or substantive associations between corn size and a linear combination of the outcome measures were observed.

Follow-up univariate analyses also indicated the effect of sex to be significantly associated with the transformed CA subscale scores (*F*_1,190_ = 6.37; *p* = 0.012); but not substantively associated with the FL or PI subscale scores. The marginal mean male transformed CA subscale score (1.062) was 0.144 points less than the marginal mean female transformed CA subscale score (1.206). A 95 % confidence interval for the difference was given by (0.032, 0.257), using the Sidak adjustment for multiple comparisons.

The analyses also indicated the effect of corn type to be substantively, but not significantly, associated with PI subscale scores (*F*_1,190_ = 3.00; *p* = 0.085); but not substantively associated with the FL or transformed CA subscale scores. The marginal mean score on the PI subscale for plantar corns (9.197) was 0.792 points greater than the marginal mean score on the PI subscale for dorsal/interdigital corns (8.406). A 95 % confidence interval for the difference was given by (−0.096, 0.161), using the Sidak adjustment for multiple comparisons.

### Analysis of self-reported pain

The mean pain score for all participants measured on the VAS was 5.29 points. A main effects univariate analysis of pain scores indicated the effect of sex to be significantly associated with pain (*F*_1,194_ = 8.73; *p* = 0.004). The marginal mean male pain score was 4.64 (SD 3.05). The marginal mean female pain score was 5.75 (2.82). Controlling for other factors and covariates, females scored 1.26 points higher on the pain score than males (95 % confidence interval: (0.42, 2.11)). The partial-η^2^ statistic of 0.015 indicated a small effect. No other statistically significant or substantive associations were observed with respect to this outcome measure.

### Analysis of QoL scores

A main effects univariate analysis of QoL scores indicated that the effect of corn type was substantively associated with quality of life (*F*_1,192_ = 3.29; *p* = 0.071). The marginal mean QoL score in those with plantar corns was 72.4 (SD 20.5). The marginal mean QoL score in those with dorsal/inter-digital corns was 78.3 (SD 17.6). Controlling for other factors and covariates, participants with dorsal/inter-digital corns scored 5.30 points higher on the QoL scale than participants with plantar corns (95 % confidence interval: (3.08, 13.7)). No other statistically significant or substantive associations were observed with respect to this outcome measure.

### Summary of significance of predictor variables

*P*-values from all controlled models are summarised in Table 2, illustrating the significance of the demographic variables of sex and age on pain and MFPDI outcomes.

**Table 2:** Significance levels associated with measured predictors in all multiple models

Outcome	Predictor			
	Sex	Age	Corn size	Corn type
MFPDI – all subscales assessed jointly	0.090	<0.001	0.382	0.114
MFPDI-FL	0.287	0.049	0.316	0.181
MFPDI-PI	0.380	<0.001	0.841	0.085
MFPDI-CA	0.012	<0.001	0.478	0.619
EQ-5D QoL	0.293	0.971	0.747	0.071
VAS Pain	0.004	0.119	0.308	0.657

### Mediation analysis

Uncontrolled regressions of FL, PI and transformed CA subscale scores of the MFPDI instrument against each of the independent variables indicated that pain (measured on the VAS scale) may act as a mediator on the casual pathway between age and each of the subscale scores; between corn type and the FI subscale scores; and between sex and the transformed CA subscale scores. However, subsequent regressions of pain on the independent variables indicated that pain was not associated with age or corn type. A regression of transformed CA subscale scores on sex and pain confirmed that pain was significantly associated with transformed CA subscale scores, controlling for sex; and that the magnitude of the parameter coefficient for sex was reduced in the presence of pain VAS scores (from 0.152 to 0.092). Hence the conditions for pain to act as a mediating variable on the casual pathway between sex and transformed CA subscale scores were satisfied.

## Discussion

The MFPDI measure, initially developed by Garrow et al. [18], has been subsequently assessed by others [1, 22, 23]. A factor structure devised from the individual items of participants studied by Menz and colleagues was found to be very close to that previously proposed by Garrow and Roddy et al. also confirmed the factor structure in a validation study of 1342 older adults.

Menz et al. [22] concluded that the MFPDI was a suitable tool for assessing foot pain in older people; however, validation of the MFPDI involved mostly middle-aged participants. Although in the current study, the mean age of participants in the sample was 59.4 years, a significant minority of participants were under 40 years, with the youngest being 19 years of age. This age distribution may have implications for the validity of the instrument applied in this particular context.

Although it is perhaps not unexpected that pain may be considered to mediate the relationship between sex and MFPDI scores, it is perhaps surprising that it is the CA (concern about appearance) scores which are so affected.

Although pain VAS scores are strongly correlated with the PI subscale scores of the MFPDI tool (*r* = 0.567; *p* < 0.001), the analysis demonstrates that different factors appear to be associated with these two measures of pain; with sex being significantly associated with pain VAS scores (women report significantly higher pain scores than men) and age being the most significant predictor of PI subscale scores (older people report significantly lower PI scores than younger people). In fact, the PI subscale of the MFPDI is more strongly correlated with the FL subscale (*r* = 0.727; *p* < 0.001) than it is with the alternative pain measure. Of all the outcome measures considered in the two halves of the analysis, the only pair which did not indicate significant correlation were EQ-5D scores and CA subscale scores (*r* = −0.192; *p* = 0.199); however, the level of substantive association between outcomes was not uniformly high.

The conclusion that age seems to have the greatest effect on foot pain as measured by the MFPDI measure was not found in the analysis of Menz et al. [22]. However, this can be ascribed to the relative homogeneity of age of participants in that study; all of whom were narrowly spread in age (mean 77.2 years; SD 4.9 years) between limits of 70 and 95 years. It is also difficult to directly compare the findings of the current analysis with that of the analysis of Roddy et al. [23]; as this was also exclusively concerned with older participants (aged 50 years and above). However, a subsequent analysis by Menz et al. [24] identified both age and sex effects, with older people and women more likely to report functional limitation effects; mirroring the findings of this current study and confirmed by previous studies [7, 8, 19]. Both age and sex may be considered proxy measurements for further unmeasured underlying causal factors, such as differences in footwear worn by men and women, and increasing age-related incidences of common co-morbidities. The understanding that informs these observations sits outside of the data set in the findings of this study. Further research may help elucidate any association to external variables influencing foot and skin mechanics leading to pain. Foot health has associated benefits to general health, social functioning and mobility [25]. Since walking and mobility is central to healthy aging, new sources of information providing evidence is necessary to develop effective public health strategies to enhance mobility in older populations [26].

### Limitations

There are a number of limitations in this study; the sample was largely drawn from participants attending podiatry services: a self-selecting group who would be assumed to be more likely to suffer with painful corns. There may be people in the general population with corns who do not experience pain and associated effects on their quality of life. This therefore precludes direct generalisability of the findings of this study from a wider population who may have corns, but are not seeking intervention. It is, however, a context-sensitive benefit to be able to generalise the findings to a wider population of those who are experiencing foot pain due to this pathology, and seeking an intervention. The sample is a reflection of those people who seek podiatric consultation in relation to foot pain, where 26 % of participant contacts are corn-related [16]. Also, footwear was not included as a variable in the current analysis, and this can be associated with the formation of corns (especially digitally) and subsequently affect mobility and quality of life. However, the inclusion of sex may be an effective proxy for footwear type due to the disparity of corn incidence reported in males and females [27].

The study did not measure corn depth, which may be a more reliable predictor of pain than the measured parameter of corn diameter, which was not found to be significantly associated with pain. This may also be related to the finding that plantar corns are more painful than dorsal/inter-digital corns; which could be ascribed to plantar pressures being exerted on the foot during walking.

## Conclusions

This study provides evidence to suggest that increasing age is significantly associated with increasing foot pain across all domains of the MFPDI instrument measured in this survey; sex and corn type show some substantive association with foot pain over individual domains; and there is no evidence to suggest that the size of a corn has any effect on disabling foot pain. The primary effect of age is of medium magnitude. Corn type has some substantive effect on quality of life; participants with dorsal/inter-digital corns report higher QoL scores than those with plantar corns. The identification of pain as a mediator suggests that sex influences levels of pain; which in turn influence CA subscale scores. The two demographic factors of age and sex seem to have an overall greater influence on the measured outcomes than clinical parameters.

### Ethical approval

This study received ethical approval from Leicestershire, Northamptonshire & Rutland Research Ethics Committee 2 (09/H0402/7) and research governance approval from the sponsor. All participants gave informed and written consent to take part.
